# Drought stress reshapes the genetic architecture of fiber quality and developmental traits in Egyptian cotton (*Gossypium barbadense* L.): a line × tester analysis under contrasting irrigation regimes

**DOI:** 10.1038/s41598-026-63583-z

**Published:** 2026-08-11

**Authors:** A. A. E. Mohamed, W. M. B. Yehia, Asmaa Hamoda, Reem M. E. Jabr, Fouad H. Saleh, Sobhi F. Lamlom

**Affiliations:** 1 Department of Agronomy, Faculty of Agriculture, Kafr El-Sheikh University, Kafr El- Sheikh, Egypt; 2 Cotton Research Institute, Agricultural Research Center (ARC), Giza, 12619 Egypt; 3 Department of Agronomy, Faculty of Agriculture, Benha University, P.O. Box 13736, Qaluobia Moshtohor, Egypt; 4 Plant Production Department, Faculty of Agriculture Saba Basha, Alexandria University, Alexandria, 21531 Egypt

**Keywords:** *Gossypium barbadense*, Drought stress, Genetic architecture, Combining ability, Gene action, Fiber development, Non-additive inheritance, Heritability, Heterosis, Climate adaptation, Genetics, Plant sciences

## Abstract

Water scarcity is an increasing threat to Egyptian cotton production, yet the mechanisms by which drought stress modifies the genetic control of fiber development and plant architecture remain poorly characterized. This study investigated how water deficit alters the genetic architecture of eight fiber quality and agronomic traits in Egyptian cotton, using a line × tester mating design comprising seven lines crossed with three testers to produce 21 F^1^ hybrids, which were evaluated under normal irrigation and drought stress at the Sakha Agricultural Experimental Research Station during the 2025 growing season. Analysis of variance partitioned genetic variation into general combining ability (GCA) and specific combining ability (SCA) components, and estimated gene action, heterosis, and heritability under both environments. Under normal irrigation, fiber quality traits micronaire, fiber length (UHML), uniformity index, and fiber strength were predominantly governed by additive genetic effects, with GCA/SCA variance ratios ranging from 13.0 to 81.6. Under drought stress, all four fiber traits showed lower GCA/SCA ratios (0.13–0.32), consistent with a relatively greater contribution of non-additive (dominance and epistatic) variance in this dataset. Narrow-sense heritability for fiber length declined from 0.980 under normal conditions to 0.206 under drought, while broad-sense heritability remained high (0.979), a pattern consistent with a relative reduction in the additive genetic component under stress. Agronomic traits were predominantly non-additive under both environments, with the notable exception of the first fruiting node under drought, which exhibited an additive-dominant ratio of 1.11, suggesting that the developmental timing of reproductive branching becomes more additively heritable under stress. Among parental genotypes, Giza 85 and Giza 92 emerged as superior donors for fiber quality under drought, while Giza 70 and Giza 87 were the most effective combiners under normal conditions. The cross Giza 85 × Giza 86 showed the highest positive mid-parent heterosis for fiber length (8.48%), uniformity index (2.01%), and fiber strength (6.57%) under drought simultaneously, suggesting complementary genetic interactions that may buffer fiber development against water deficit. These findings suggest that drought is associated with a shift in the relative contribution of additive and non-additive genetic control over fiber quality and developmental traits in this population and environment, with potential implications for climate-resilient cotton breeding strategy. Hybridization-based approaches exploiting non-additive variance may be worth considering for drought-targeted programs, whereas pedigree selection appears more appropriate under favorable conditions; confirmation across additional environments and germplasm would strengthen these recommendations.

**Supplementary Information:**

The online version contains supplementary material available at 10.1038/s41598-026-63583-z.

## Introduction

*Gossypium barbadense* L. (Egyptian cotton) occupies a distinctive niche in global textile markets by virtue of its extra-long staple fibers, exceptional fineness, and high fiber strength properties that command premium prices and cannot be replicated by *G. hirsutum* or synthetic alternatives^1,2^. Egypt has historically been the primary producer of extra-long staple cotton, supplying specialty fiber for high-end spinning operations worldwide. However, this production system faces mounting pressures from climate change, most acutely through increasing water scarcity in the Nile Delta region, where the majority of Egyptian cotton is cultivated^2,3^. Projected reductions in irrigation water availability, combined with rising temperatures and more frequent drought events, threaten both the productivity and fiber quality of Egyptian cotton, creating an urgent need to understand how these genotypes respond to water deficit at the genetic level.

Drought stress influences cotton growth and development through multiple overlapping mechanisms. Water deficit during critical developmental windows suppresses cell elongation by reducing turgor pressure, thereby directly constraining fiber elongation during the primary wall synthesis phase^4–6^. Secondary cell wall deposition, which determines fiber strength and micronaire value through cellulose accumulation, is sensitive to carbon allocation patterns that are disrupted under stress through stomatal closure and reduced photosynthesis^7^. At the whole-plant level, drought alters the hormonal balance that governs reproductive development. Elevated abscisic acid (ABA) concentrations under water deficit are associated with earlier stomatal closure, altered auxin-gibberellin interactions, and changes in the timing of fruiting branch initiation^8,9^. These physiological responses collectively alter trait expression across fiber quality, plant architecture, and reproductive transition, potentially in genotype-dependent ways.

Understanding the genetic architecture of target traits under stress is central to developing effective breeding strategies. The relative contributions of additive and non-additive gene action determine whether traits respond to selection in early generations or require exploitation through heterosis in hybrid programs^10–12^. Drought stress is known to modify this balance: conditions that amplify non-additive genetic effects, particularly dominance and epistasis, reduce the effectiveness of early-generation selection while increasing the potential value of F^1^ hybrid development^13,14^. Characterizing these shifts requires the explicit evaluation of combining ability, heritability, and heterosis under target stress conditions, as estimates derived from well-irrigated trials may be unreliable guides for drought breeding^2,15^.

The line × tester (L × T) mating design provides a systematic framework for partitioning genetic variation into general combining ability (GCA) effects, reflecting primarily additive gene action, and the average performance of a parent across all crosses, and specific combining ability (SCA) effects, reflecting non-additive interactions between specific parental combinations^16–18^. Prior applications of L × T analysis in Egyptian cotton have documented the importance of both additive and non-additive gene action for fiber and yield traits under normal conditions^19–22^. Comparatively little is known about how drought stress modifies GCA rankings, SCA patterns, heritability, or heterosis for the traits most critical to fiber quality and agronomic adaptation. This knowledge gap is particularly consequential, given that the Nile Delta cotton-growing region is experiencing more frequent drought episodes. The present study differs from these previous Egyptian cotton combining-ability reports in that it evaluates the same set of parents and crosses under both normal irrigation and a controlled drought treatment within a single trial, allowing GCA, SCA, heterosis, and heritability estimates to be compared directly between environments rather than inferred separately from studies conducted under different conditions, seasons, or genetic materials.

The present study was designed to address this gap by: (i) quantifying how drought stress alters the balance between additive and non-additive genetic control of fiber quality traits micronaire, fiber length, uniformity index, and fiber strength and agronomic traits first fruiting node, number of vegetative branches, number of fruiting branches, and plant height; (ii) identifying parental genotypes whose combining ability profiles are robust or adaptive under drought; (iii) estimating heterosis under both environments to assess hybrid potential under stress; and (iv) deriving heritability estimates that guide selection strategy decisions for each environment. The central hypothesis is that drought stress induces a systematic shift in the genetic architecture of fiber quality traits from predominantly additive to predominantly non-additive control, with implications for trait expression mechanisms, inheritance patterns, and breeding program design.

## Materials and methods

### Plant material

Ten Egyptian cotton genotypes were used as parental material, divided into seven lines (L1–L7) and three testers (T1–T3). Lines comprised Giza 70 (L1), Giza 85 (L2), Giza 87 (L3), Giza 92 (L4), Giza 93 (L5), Giza 96 (L6), and Giza 89 × Giza 86 (L7). Testers were Giza 95 (T1), Giza 86 (T2), and Giza 94 (T3). All genotypes were obtained from the Cotton Research Institute (CRI), Agricultural Research Center (ARC), Giza, Egypt. The parental set represented a broad range of genetic backgrounds, encompassing five extra-long staple varieties (Giza 70, Giza 87, Giza 92, Giza 93, and Giza 96) and five long-staple varieties (Giza 85, Giza 89 × Giza 86, Giza 95, Giza 86, and Giza 94), ensuring a wide spectrum of allelic diversity for the traits of interest (Table 1).

**Table 1:** Pedigree, origin, and staple category of the ten parental Egyptian cotton genotypes used in the line × tester mating design.

No.	Genotypes	Origin	Pedigree	Category
Lines				
L1	Giza 70	Egypt	Giza 59 A × Giza 51B	Extra-long
L2	Giza 85	Egypt	Giza 67 × CB 58	Long staple
L3	Giza 87	Egypt	(Giza 77 × Giza 45) A	Extra-long
L4	Giza 92	Egypt	Giza 84 × (Giza 74 × Giza 68)	Extra-long
L5	Giza 93	Egypt	Giza 77 × S6	Extra-long
L6	Giza 96	Egypt	Giza 84 × (Giza 70 × Giza 51b) × S62	Extra-long
L7	Giza 89 × Giza 86	Egypt	(Giza 75 × 6022) × (Giza 75 × Giza 81)	Long staple
**Testers**				
T1	Giza 95	Egypt	(Giza 83 × (Giza 75 × 8544)) × Giza 80	Long staple
T2	Giza 86	Egypt	Giza 75 × Giza 81	Long staple
T3	Giza 94	Egypt	10,229 × Giza 86	Long staple

### Crossing program and experimental design

A complete L × T mating design was implemented during the 2024 growing season at the Sakha Agricultural Experimental Research Station, Kafr El-Sheikh Governorate, Egypt (31°07’N, 30°57’E, approximately 6 m above sea level). Each of the seven lines was crossed with each of the three testers to generate 21 F^1^ hybrids by hand emasculation and pollination.

The ten parental genotypes and 21 F^1^ hybrids were evaluated during the 2025 growing season under two water management treatments: (i) normal irrigation water applied according to the standard production schedule for the Nile Delta; and (ii) drought stress irrigation withheld during the boll development period to create water deficit. Under normal irrigation, plants received water according to the recommended schedule for cotton in the Nile Delta, with irrigation applied at critical growth stages so that no water limitation occurred during vegetative growth, flowering, or boll development; this treatment received 8–10 irrigations over the growing season, sufficient to meet crop evapotranspiration requirements. For the drought treatment, irrigation was restricted to three applications for the entire season, beginning from early growth and continuing through reproductive development; the three irrigations were timed to ensure plant establishment and survival while imposing sustained water deficit, representing an approximately 60–70% reduction in total water supply relative to the normal-irrigation treatment. Both treatments received identical fertilization, pest control, and weed management according to the recommendations of the Cotton Research Institute. A randomized complete block design (RCBD) with three replications was employed for each treatment; three replications is standard practice for line × tester field evaluations of this size (21 hybrids plus 10 parents) and balances statistical power against the land and labor required for hand emasculation and pollination of this many crosses. Each experimental unit consisted of a single row 5 m in length, with 0.7 m between-row spacing and 0.4 m spacing between hills within rows; hills were thinned to two plants shortly after emergence. Physicochemical properties of the experimental soil and prevailing air temperature during the growing seasons are given in Tables S1 and S2.

### Trait measurement

#### Fiber quality traits

Fiber quality analysis was conducted at the Cotton Technology Research Division Laboratories, CRI, ARC, Giza, using a High-Volume Instrument (HVI) system on seed cotton samples harvested at full maturity. Four parameters were measured: micronaire (FF), an indirect measure of fiber fineness and maturity via airflow resistance; upper half mean length (UHML, mm), the mean length of the longer 50% of fibers, the primary staple-length parameter; uniformity index (UI, %), the ratio of mean fiber length to UHML, reflecting within-sample length consistency; and fiber strength (FS, g/tex), bundle strength measured by the HVI pressley method.

#### Agronomic traits

Four agronomic traits were recorded from five representative competitive plants per row: first fruiting node (FFN) position of the first fruiting branch on the main stem, counted from the cotyledon node; number of vegetative branches (NVB); number of fruiting branches (NFB); and plant height (PH, cm) measured at physiological maturity.

### Statistical analysis

Analysis of variance was performed separately for each trait and each growing environment (normal and drought) using the standard RCBD model. Genotypic mean squares were then partitioned into components attributable to Lines (GCA of lines), Testers (GCA of testers), and Lines × Testers interaction (SCA) following the procedure described by Kempthorne (1957). The significance of mean squares was tested using the F-ratio at the following probability levels: *P* ≤ 0.05 (*), *P* ≤ 0.01 (**), and *P* ≤ 0.001 (***). Least significant difference (LSD) values were calculated at the 5% probability level for pairwise comparisons of genotype means, according to Steel and Torrie (1980).

General combining ability (GCA) effects for lines (*ĝ*_i_) and testers (*ĝ*_j_) were estimated as deviations of the respective parental mean cross performance from the overall grand mean of all crosses. Specific combining ability (SCA) effects for individual crosses (*ŝ*_ij_) were estimated as deviations of individual cross performance from the grand mean after removal of GCA effects. Variance components for GCA (σ²_GCA_) and SCA (σ²_SCA_) were derived from the mean squares of Lines, Testers, and Lines × Testers according to the expectations of Kempthorne (1957), and their ratio (σ²_GCA_/σ²_SCA_) was used as an index of the relative importance of additive (GCA ratio > 1.0) versus non-additive (GCA ratio < 1.0) gene action. Significance of individual GCA and SCA effects was tested by a t-test against the appropriate error mean square. Because variance components are estimated from mean squares, sampling error can produce a negative estimate when the observed mean square falls below the error mean square; such estimates are not biologically interpretable as negative variances. Following standard practice, any negative σ²_GCA_ or σ²_SCA_ estimate was set to zero prior to calculating the GCA/SCA ratio. Under this convention, a ratio of zero indicates that the GCA component was non-significantly different from zero relative to SCA, whereas a ratio denoted as undefined (where σ²_SCA_ was set to zero) indicates that the SCA component was non-significantly different from zero relative to GCA; both cases are noted explicitly in Table 2 rather than reported as raw negative ratios. The additive genetic variance (V_A_) used to derive narrow-sense heritability was obtained from the GCA variance component according to the standard line × tester relationship, σ²_GCA_ = ¼V_A_ (pooled over lines and testers), so that V_A_ = 4σ²_GCA_, consistent with Kempthorne (1957) and Singh and Chaudhary (1985).

**Table 2:** GCA/SCA variance ratios and predominant gene action under normal and drought conditions.

Trait	Environment	σ²GCA	σ²SCA	GCA/SCA Ratio	Predominant Gene Action
MIC (FF)	Normal	0.0929	0.0071	13.033	Additive
	Drought	0.0290	0.1330	0.218	Non-additive
FL (mm)	Normal	0.5461	0.0067	81.627	Additive
	Drought	0.1199	0.9099	0.132	Non-additive
UI (%)	Normal	0.1283	0.0067	19.236	Additive
	Drought	0.0824	0.2610	0.316	Non-additive
FS (g/tex)	Normal	0.0992	-0.0003	Undefined (σ²SCA = 0)	Additive (complete; σ²SCA ≈ 0)
	Drought	-0.0064	0.0884	0.000	Non-additive (σ²GCA = 0)
FFN	Normal	-0.0060	0.4155	0.000	Non-additive (σ²GCA = 0)
	Drought	0.1766	0.1591	1.11	Additive-dominant
NVB	Normal	-0.0794	0.7798	0.000	Non-additive (σ²GCA = 0)
	Drought	0.0099	0.2226	0.045	Non-additive
NFB	Normal	4.2817	7.6611	0.559	Non-additive
	Drought	-0.3274	4.0433	0.000	Non-additive (σ²GCA = 0)
PH (cm)	Normal	85.8135	296.0913	0.29	Non-additive
	Drought	8.5476	87.2885	0.098	Non-additive

Ratio < 1.0 = non-additive; 1.0–2.0 = Additive-dominant; >2.0 = Additive. MIC = Micronaire (FF); FL = Fiber Length (UHML mm); UI% = Uniformity Index; FS = Fiber Strength (g/tex); FFN = First Fruiting Node; NVB = No. Vegetative Branches; NFB = No. Fruiting Branches; PH = Plant Height (cm). GCA = General Combining Ability; SCA = Specific Combining Ability. * *P* ≤ 0.05; ** *P* ≤ 0.01; *** *P* ≤ 0.001.

Mid-parent heterosis (HMP) was defined as the percentage deviation of the F^1^ cross mean performance from the arithmetic mean of its two parents, and was calculated as:

$$\:\mathrm{H}\mathrm{M}\mathrm{P}\:\left(\mathrm{\%}\right)\:=\:\left[\right(\mathrm{F}1\hspace{0.17em}-\hspace{0.17em}\mathrm{M}\mathrm{P})\:/\:\mathrm{M}\mathrm{P}]\:\times\:\:100$$

where MP = (P_1_ + P_2_) / 2, P_1_ is the line mean, and P_2_ is the tester mean.

Better-parent heterosis (HBP), also termed heterobeltiosis, was defined as the percentage deviation of the F^1^ cross mean from the superior parent value, and was calculated as:

$$\:\mathrm{H}\mathrm{B}\mathrm{P}\:\left(\mathrm{\%}\right)\:=\:\left[\right(\mathrm{F}1\hspace{0.17em}-\hspace{0.17em}\mathrm{B}\mathrm{P})\:/\:\mathrm{B}\mathrm{P}]\:\times\:\:100$$

where BP is the mean of the better parent (whichever parent had the higher value for the trait). The significance of heterosis estimates was tested using the LSD method at *P* ≤ 0.05 and *P* ≤ 0.01 probability levels, according to Steel and Torrie^23^.

Broad-sense heritability (H²_b_) was estimated as the ratio of total genotypic variance (V_G_) to total phenotypic variance (V_P_), where V_G_ = (MS_G_ − MS_e_) / r, V_P_ = V_G_ + MS_e_, MS_G_ is the genotype mean square from ANOVA, MS_e_ is the error mean square, and r is the number of replications. Narrow-sense heritability (H²_n_) was estimated as the ratio of additive genetic variance (V_A_) to total phenotypic variance (V_P_), where V_A_ was derived from the GCA variance component using the relationships established by Kempthorne^24^. Heritability formulas followed the procedures described by Allard^25^ and Mather^26^.

All statistical analyses were performed using R version 4.5 (R Core Team, 2025) with the readxl, dplyr, tidyr, and FactoMineR packages for data processing and multivariate analysis. Analysis of variance, GCA and SCA estimation, heterosis calculations, and heritability derivations were implemented using custom R scripts.

## Results

### Analysis of variance: genetic variation across environments

Variance analysis revealed highly significant genetic variation for all eight traits under both irrigation treatments (Table 3). Under normal conditions, mean squares for Lines (GCA) were highly significant for all four fiber quality traits and for the number of fruiting branches and plant height, indicating that parental genotypes differ substantially in their additive contributions to these traits. Testers (GCA) were similarly highly significant for all fiber traits and most agronomic traits. The Lines × Testers (SCA) interaction was significant for micronaire, fiber length, and uniformity index, but not for fiber strength, and highly significant for all four agronomic traits, indicating that specific parental combinations produced trait expression that deviated from additive expectations, particularly for plant architecture and developmental timing.

**Table 3:** Mean squares from the line × tester ANOVA for fiber quality and agronomic traits under normal and drought conditions.

Source of Variation	df	MIC (FF)	FL (mm)	UI (%)	FS (g/tex)	FFN	NVB	NFB	PH (cm)
Normal Conditions									
Replications	2	0.013	0.010	0.008	0.003	0.048	0.000	0.762	36.012
Crosses	20	0.467	2.656	0.622	0.470	1.575	2.393	48.986	1376.071
Lines (GCA)	6	0.639***	4.999***	0.815***	0.732***	1.333**	1.143	89.202***	2434.821***
Testers (GCA)	2	2.532***	11.377***	3.590***	2.474***	2.036**	3.464**	61.179***	970.536***
Lines × Testers (SCA)	12	0.036*	0.031**	0.031**	0.004	1.619***	2.839***	26.845***	914.286***
Error	40	0.015	0.011	0.011	0.005	0.373	0.500	3.862	26.012
Total	62	-	-	-	-	-	-	-	-
**Drought Conditions**									
Replications	2	0.009	0.048	0.010	0.010	0.298	0.107	0.429	149.333
Crosses	20	0.573	3.479	1.261	0.249	1.718	1.075	14.061	376.993
Lines (GCA)	6	1.039***	6.075***	2.607***	0.271***	4.143***	1.500**	13.369**	420.810***
Testers (GCA)	2	0.159***	0.041	0.023	0.018	0.250	0.250	7.000	495.107**
Lines × Testers (SCA)	12	0.410***	2.754***	0.794***	0.276***	0.750**	1.000**	15.583***	335.399***
Error	40	0.011	0.024	0.011	0.011	0.273	0.332	3.454	73.533
Total	62	-	-	-	-	-	-	-	-

* *P* ≤ 0.05; ** *P* ≤ 0.01; *** *P* ≤ 0.001. Abbreviations: MIC=Micronaire; FL=Fiber Length; UI=Uniformity Index; FS=Fiber Strength; FFN=First Fruiting Node; NVB = No. Vegetative Branches; NFB = No. Fruiting Branches; PH=Plant Height.

Under drought stress, the pattern of significance shifted in ways consistent with environmental modification of genetic control. Lines (GCA) remained highly significant for all traits, confirming that parental additive effects are detectable under stress. However, Tester (GCA) effects under drought were significant only for micronaire and plant height, a notable contraction from normal conditions, when tester GCA was significant for all fiber traits, suggesting that the genetic contributions of testers are more environment-dependent than those of lines. The Lines × Testers (SCA) interaction was highly significant for all eight traits under drought, including fiber strength (which had been non-significant under normal conditions), indicating that drought substantially amplified non-additive gene interactions across traits.

### Drought-induced shifts in gene action (σ²GCA/σ²SCA Ratios)

The σ²GCA/σ²SCA ratios indicate an environment-dependent shift in the relative contributions of additive and non-additive gene action, particularly for fiber quality traits (Table 2). Under normal irrigation, fiber quality traits appeared predominantly under additive genetic control: fiber length showed the highest ratio (81.6), followed by uniformity index (19.2) and micronaire (13.0). These values suggest that parental GCA effects outweighed specific cross interactions for fiber traits under favorable water availability, a pattern broadly consistent with classical quantitative genetics theory for traits under stabilizing selection. For fiber strength under normal conditions, the SCA variance component was negative and was treated as zero following standard convention, yielding an undefined GCA/SCA ratio that is reported in Table 2 rather than as a raw negative value; this pattern is consistent with predominantly additive control for this trait under normal conditions.

Under drought stress, the GCA/SCA ratios for the other three fiber quality traits declined below unity: fiber length (0.13), uniformity index (0.32), and micronaire (0.22). For fiber strength, the GCA variance component was negative and was treated as zero following the convention described in Methods, yielding a ratio of zero that is classified as non-additive. This reconciles the apparent conflict between Tables 3 and 2 for this trait: the Lines and Lines × Testers mean squares for fiber strength under drought are both highly significant in Table 3 because the F-test compares each mean square directly against the error mean square, whereas the Table 2 variance component is calculated by subtracting the error mean square from the Lines × Testers mean square; when that difference is small or negative relative to sampling error, the resulting σ²GCA estimate can be near zero or negative even though the corresponding mean square is statistically significant. This pattern suggests a relatively greater contribution of dominance and epistatic variance to fiber quality expression under drought, with a correspondingly smaller relative contribution from additive allelic differences; because GCA variance can also include additive × additive epistasis and SCA variance reflects dominance, this ratio should be interpreted as reflecting the relative magnitude of these variance components rather than as evidence of a qualitative switch in the underlying molecular control of the trait. Traits that respond predictably to additive genetic differences under optimal conditions may therefore show a relatively greater influence of specific-cross (non-additive) effects when water supply is limiting.

For agronomic traits, non-additive gene action predominated under both environments. Plant height (0.29 normal; 0.10 drought) was non-additively controlled in both environments. Number of fruiting branches was likewise classified as non-additive in both environments (ratio 0.56 under normal conditions; under drought the GCA variance component was negative and was treated as zero, yielding a ratio of zero), consistent with the complex polygenic architecture of these traits and their sensitivity to environmental variation. A notable exception was the first fruiting node under drought, which showed an additive-dominant ratio of 1.11, the only trait to show predominantly additive control under water stress. This pattern may reflect that the developmental timing of fruiting branch initiation, a determinant of crop earliness, is under relatively greater additive genetic influence under stress conditions in this dataset, though this interpretation would benefit from confirmation in additional environments.

### General combining ability effects

#### Under normal irrigation

Under normal conditions, Giza 70 was a broadly favorable combiner for fiber quality, contributing significant positive GCA for fiber length (0.60), uniformity index (0.39), and fiber strength (0.38), while Giza 87 showed a highly significant negative GCA for micronaire (− 0.53), a desirable direction alongside significant positive effects for fiber length (0.60) and fiber strength (0.38). Giza 93 contributed significant positive GCA for fiber length (0.81) and uniformity index (0.30). In contrast, Giza 96 (fiber length: −0.22; fiber strength: −0.30) and Giza 89 × Giza 86 (fiber length: −1.28; uniformity index: −0.30) were consistently unfavorable fiber-quality combiners under normal conditions. Among testers, Giza 45 was the most broadly useful parent, contributing significant negative GCA for micronaire (− 0.40) and positive GCA for fiber length (0.79), uniformity index (0.48), fiber strength (0.29), vegetative branches (0.45), fruiting branches (1.86), and plant height (6.55). Giza 86 and Giza 94 showed negative GCA for fiber length and fiber quality traits, limiting their utility as fiber-improvement parents under normal conditions (Table 4).

**Table 4:** General combining ability (GCA) effects for lines and testers under normal and drought conditions.

Genotype	MIC (FF)	FL (mm)	UI (%)	FS (g/tex)	FFN	NVB	NFB	PH (cm)
Normal Conditions								
Lines								
Giza 70	0.021	0.601***	0.387***	0.380***	-0.333	-0.286	-1.024	12.738***
Giza 85	0.102*	-0.518***	-0.331***	-0.288***	0.333	-0.286	-3.690***	-24.762***
Giza 87	-0.532***	0.598***	-0.266***	0.377***	0.333	0.214	2.976***	17.738***
Giza 92	0.081	0.000	0.042	-0.065*	0.500*	0.048	-0.690	-7.262***
Giza 93	-0.116**	0.814***	0.301***	0.044	-0.500*	0.548*	-2.690***	-12.262***
Giza 96	0.303***	-0.217***	0.170***	-0.298***	-0.167	0.214	-0.190	-3.929*
G.89×G.86	0.141**	-1.279***	-0.303***	-0.150***	-0.167	-0.452	5.310***	17.738***
Testers								
Giza 45	-0.401***	0.791***	0.475***	0.292***	0.214	0.452**	1.857***	6.548***
Giza 94	0.205***	-0.127***	-0.197***	-0.378***	-0.357*	-0.119	-0.357	0.476
Giza 86	0.196***	-0.665***	-0.278***	0.086***	0.143	-0.333*	-1.500**	-7.024***
LSD₀.₀₅	0.116	0.099	0.101	0.069	0.582	0.674	1.872	4.859
Drought Conditions								
*Lines*								
Giza 70	-0.667***	0.506***	0.279***	0.074*	-0.619***	-0.500*	-0.524	8.619**
Giza 85	-0.230***	1.077***	0.783***	0.228***	-0.952***	-0.500*	-1.690**	-11.881***
Giza 87	0.098**	-0.129*	0.111**	-0.060	0.881***	0.333	1.643*	-0.381
Giza 92	0.170***	0.760***	0.217***	0.178***	0.548**	0.500*	0.643	3.619
Giza 93	0.298***	-0.251***	0.005	-0.016	-0.119	0.000	0.643	4.452
Giza 96	0.086*	-1.141***	-0.785***	-0.256***	0.548**	-0.167	0.643	1.119
G.89×G.86	0.244***	-0.822***	-0.610***	-0.148***	-0.286	0.333	-1.357*	-5.548
*Testers*								
Giza 45	-0.092***	0.031	0.030	-0.015	0.119	-0.095	0.667	1.500
Giza 94	0.011	0.020	0.005	-0.019	-0.095	-0.024	-0.333	3.929*
Giza 86	0.081***	-0.051	-0.035	0.034	-0.024	0.119	-0.333	-5.429**
LSD₀.₀₅	0.100	0.148	0.099	0.099	0.497	0.549	1.771	8.170

* *P* ≤ 0.05; ** *P* ≤ 0.01; *** *P* ≤ 0.001.MIC = Micronaire (FF); FL = Fiber Length (UHML mm); UI% = Uniformity Index; FS = Fiber Strength (g/tex); FFN = First Fruiting Node; NVB = No. Vegetative Branches; NFB = No. Fruiting Branches; PH = Plant Height (cm). GCA = General Combining Ability; SCA = Specific Combining Ability. * *P* ≤ 0.05; ** *P* ≤ 0.01; *** *P* ≤ 0.001.

For agronomic traits, Giza 87 and Giza 89 × Giza 86 contributed significant positive GCA for fruiting branches and plant height, while Giza 85 had the most strongly negative GCA for plant height (− 24.76) and fruiting branches (− 3.69), indicating that its progeny tend toward compact, reduced-branch phenotypes under well-watered conditions.

#### Under drought stress

Drought stress substantially reorganized GCA rankings, indicating environment-dependent expression of parental allelic effects. Giza 85 emerged as the superior fiber-length combiner under drought (GCA = 1.08, *P* ≤ 0.001), a substantial shift from its negative or neutral fiber-length GCA under normal conditions, alongside favorable effects on uniformity index (0.78) and fiber strength (0.23). Giza 92 maintained consistent positive GCA for all four fiber quality traits under drought (micronaire: 0.17; fiber length: 0.76; uniformity index: 0.22; fiber strength: 0.18), suggesting that its complex pedigree (Giza 84 × (Giza 74 × Giza 68)) may incorporate drought-adaptive alleles that maintain fiber quality under water deficit. Giza 70 showed a favorable negative GCA for micronaire (− 0.67) under drought, alongside positive GCA for fiber length (0.51). Giza 96 remained an unfavorable fiber-quality combiner under drought (fiber length: −1.14; uniformity index: −0.79; fiber strength: −0.26), as did Giza 89 × Giza 86 (Table 4).

For agronomic traits under drought, Giza 87 showed significant positive GCA for the first fruiting node (0.88) and fruiting branches (1.64), whereas Giza 85 had the most negative GCA for the first fruiting node (− 0.95). Tester effects under drought were substantially diminished compared to normal conditions, with Giza 45 retaining only a modestly significant negative GCA for micronaire (− 0.09), consistent with the overall reduction in tester GCA significance under stress documented in the ANOVA.

### Specific combining ability effects

#### Under normal irrigation

SCA effects under normal conditions were generally small for fiber quality traits relative to GCA effects, consistent with the high GCA/SCA ratios in this environment. The most notable exception was Giza 96 × Giza 45, which showed highly significant positive SCA for micronaire (0.262), fiber length (0.253), and uniformity index (0.253) simultaneously, a combination of complementary non-additive interactions that partially compensated for Giza 96’s unfavorable fiber-quality GCA. For agronomic traits, Giza 85 × Giza 45 produced the largest positive SCA for plant height (35.12), while Giza 93 × Giza 45 showed strongly negative SCA for fruiting branches (− 4.02) and plant height (− 17.38), suggesting suppressive interactions despite acceptable parental GCA values. Giza 93 × Giza 86 showed the strongest positive SCA for fruiting branches (4.33) and plant height (13.69) under normal conditions (Table 5).

**Table 5:** Specific Combining Ability (SCA) Effects Under Normal Conditions.

Cross	MIC (FF)	FL (mm)	UI (%)	FS (g/tex)	FFN	NVB	NFB	PH (cm)
Giza 45 ×								
Giza 70 × Giza 45	-0.03	-0.03	-0.03	-0.002	0.119	1.21**	-2.69*	2.62
Giza 85 × Giza 45	-0.08	-0.09	-0.09	-0.05	-0.048	-0.28	1.97	35.12***
Giza 87 × Giza 45	-0.02	-0.02	-0.02	0.008	0.452	-0.28	-0.19	-2.38
Giza 92 × Giza 45	-0.07	-0.01	-0.02	0.014	0.78*	0.38	-0.52	-7.38*
Giza 93 × Giza 45	-0.03	-0.04	-0.04	-0.005	-0.71*	-0.12	-4.02**	-17.38***
Giza 96 × Giza 45	0.26***	0.25***	0.25***	0.064	-0.54	-1.28**	2.97*	-5.71
G.89×G.86 × Giza 45	-0.05	-0.06	-0.05	-0.025	-0.04	0.38	2.47*	-4.88
***Giza 94 ×***								
Giza 70 × Giza 94	0.005	0.02	0.023	0.005	-0.31	-0.71	1.52	-11.31***
Giza 85 × Giza 94	0.04	0.05	0.05	0.04	-0.47	-0.71	-2.81*	-23.81***
Giza 87 × Giza 94	0.02	0.03	0.037	0.02	-0.47	1.28**	1.02	-1.31
Giza 92 × Giza 94	0.11	0.02	0.02	0.00	0.85*	-0.04	1.19	3.69
Giza 93 × Giza 94	0.02	0.04	0.04	0.021	0.35	-0.54	-0.31	3.69
Giza 96 × Giza 94	-0.17*	-0.15*	-0.15*	-0.05	0.02	0.28	1.69	22.86***
G.89×G.86 × Giza 94	-0.02	-0.01	-0.03	-0.02	0.02	0.45	-2.3*	6.19*
***Giza 86 ×***								
Giza 70 × Giza 86	0.02	0.02	0.02	-0.003	0.19	-0.50	1.16	8.69**
Giza 85 × Giza 86	0.040	0.03	0.03	0.02	0.52	1.00*	0.83	-11.31***
Giza 87 × Giza 86	-0.002	-0.01	-0.02	-0.03	0.02	-1.00*	-0.83	3.69
Giza 92 × Giza 86	-0.04	-0.01	0.00	-0.014	-1.64***	-0.33	-0.66	3.69
Giza 93 × Giza 86	0.008	0.001	-0.002	-0.016	0.35	0.66	4.33***	13.69***
Giza 96 × Giza 86	-0.09	-0.10	-0.10	-0.007	0.52	1.00*	-4.67***	-17.14***
G.89×G.86 × Giza 86	0.07	0.07	0.07	0.05	0.02	-0.83*	-0.17	-1.30
**LSD₀.₀₅**	**0.20**	**0.17**	**0.17**	**0.12**	**1.007**	**1.16**	**3.24**	**8.41**

* *P* ≤ 0.05; ** *P* ≤ 0.01; *** *P* ≤ 0.001.MIC = Micronaire (FF); FL = Fiber Length (UHML mm); UI% = Uniformity Index; FS = Fiber Strength (g/tex); FFN = First Fruiting Node; NVB = No. Vegetative Branches; NFB = No. Fruiting Branches; PH = Plant Height (cm). GCA = General Combining Ability; SCA = Specific Combining Ability. * *P* ≤ 0.05; ** *P* ≤ 0.01; *** *P* ≤ 0.001.

#### Under drought stress

Under drought (Table 6), SCA patterns diverged substantially from normal conditions. Giza 93 × Giza 45 showed the highest positive SCA for fiber length (1.427) and uniformity index (0.777), values considerably larger than any SCA effect for fiber length observed under normal conditions. This cross, combining a line with above-average drought GCA (Giza 93 had positive fiber-length GCA under normal conditions) and the most broadly useful tester, appears to exhibit particularly favorable epistatic interactions for fiber-length maintenance under water deficit. Giza 85 × Giza 86 showed significant positive SCA for fiber length (0.654), uniformity index (0.638), and fiber strength (0.286) under drought. Conversely, Giza 87 × Giza 45, which showed neutral SCA under normal conditions, exhibited strongly negative drought SCA for fiber length (− 1.096) and micronaire (− 0.423), indicating suppressive cross-specific interactions that impair fiber quality under stress. Giza 92 × Giza 45 showed a highly significant positive SCA for the first fruiting node (0.881) under drought, consistent with earlier fruiting branch initiation in this combination under stress.

**Table 6:** Specific Combining Ability (SCA) Effects Under Drought Conditions.

Cross	MIC (FF)	FL (mm)	UI (%)	FS (g/tex)	FFN	NVB	NFB	PH (cm)
Giza 45 ×								
Giza 70 × Giza 45	0.196**	0.173	0.357***	0.219***	0.548	-0.071	3.667**	10.167*
Giza 85 × Giza 45	0.079	-0.978***	-0.327***	-0.315***	-0.119	-0.071	-1.667	-20.833***
Giza 87 × Giza 45	-0.423***	-1.096***	-0.478***	-0.200**	-0.452	0.095	2.000	6.667
Giza 92 × Giza 45	-0.345***	-0.034	-0.334***	0.162*	0.881**	-0.571	1.000	7.667
Giza 93 × Giza 45	0.227***	1.427***	0.777***	0.156*	-0.452	0.429	-0.500	-5.667
Giza 96 × Giza 45	0.336***	-0.237*	-0.036	-0.207**	-0.119	0.595	-3.000**	-4.833
G.89×G.86 × Giza 45	-0.071	0.744***	0.040	0.185**	-0.286	-0.405	-1.500	6.833
***Giza 94 ×***								
Giza 70 × Giza 94	0.273***	-0.485***	-0.137*	-0.396***	-0.238	0.357	-1.833	-4.762
Giza 85 × Giza 94	-0.334***	0.324***	-0.311***	0.029	0.095	-0.143	0.333	15.738**
Giza 87 × Giza 94	0.115	0.806***	0.438***	0.395***	0.262	0.024	-2.500*	-5.262
Giza 92 × Giza 94	0.342***	-0.683***	0.132*	-0.244***	-0.905**	-0.143	-1.000	-12.262*
Giza 93 × Giza 94	0.055	-0.982***	-0.467***	-0.210**	0.262	0.357	1.500	4.405
Giza 96 × Giza 94	-0.543***	0.398***	-0.237***	0.320***	0.095	-0.976**	3.000**	2.738
G.89×G.86 × Giza 94	0.092	0.623***	0.582***	0.106	0.429	0.524	0.500	-0.595
***Giza 86 ×***								
Giza 70 × Giza 86	-0.470***	0.312**	-0.221***	0.177**	-0.310	-0.286	-1.833	-5.405
Giza 85 × Giza 86	0.256***	0.654***	0.638***	0.286***	0.024	0.214	1.333	5.095
Giza 87 × Giza 86	0.308***	0.290**	0.040	-0.195**	0.190	-0.119	0.500	-1.405
Giza 92 × Giza 86	0.002	0.717***	0.202**	0.083	0.024	0.714*	0.000	4.595
Giza 93 × Giza 86	-0.282***	-0.445***	-0.311***	0.054	0.190	-0.786*	-1.000	1.262
Giza 96 × Giza 86	0.207**	-0.161	0.273***	-0.113	0.024	0.381	0.000	2.095
G.89×G.86 × Giza 86	-0.021	-1.367***	-0.622***	-0.291***	-0.143	-0.119	1.000	-6.238
**LSD₀.₀₅**	**0.172**	**0.256**	**0.171**	**0.171**	**0.862**	**0.951**	**3.067**	**14.151**

* *P* ≤ 0.05; ** *P* ≤ 0.01; *** *P* ≤ 0.001.MIC = Micronaire (FF); FL = Fiber Length (UHML mm); UI% = Uniformity Index; FS = Fiber Strength (g/tex); FFN = First Fruiting Node; NVB = No. Vegetative Branches; NFB = No. Fruiting Branches; PH = Plant Height (cm). GCA = General Combining Ability; SCA = Specific Combining Ability. * *P* ≤ 0.05; ** *P* ≤ 0.01; *** *P* ≤ 0.001.

### GCA/SCA variance ratio and predominant gene action

The ratio of GCA to SCA variance (σ²GCA/σ²SCA), computed from the combined analysis with negative variance-component estimates set to zero, indicated predominantly non-additive gene action for 6 of the 8 traits and predominantly additive gene action for 2 traits when data from both environments were pooled (Table 7). Fiber strength and number of fruiting branches showed ratios above unity, indicating a relatively larger additive contribution for these two traits even after pooling across the contrasting irrigation regimes, whereas micronaire, fiber length, uniformity index, first fruiting node, number of vegetative branches, and plant height were classified as non-additive on a combined basis. This combined-analysis classification reflects the average gene action across both environments and should be interpreted alongside the significant Lines × Environment, Testers × Environment, and Lines × Testers × Environment interactions reported in Table 1, which indicate that the relative importance of additive and non-additive effects differed between the normal-irrigation and drought environments for every trait.

**Table 7:** GCA/SCA variance ratio and predominant gene action for eight fiber-quality and agronomic traits, combined analysis across normal-irrigation and drought environments.

Trait	σ²GCA	σ²SCA	GCA/SCA ratio	Predominant gene action
MIC	0.0333	0.0431	0.772	Non-additive
FL	0.2072	0.2211	0.937	Non-additive
UI	0.0513	0.0639	0.802	Non-additive
FS	0.0253	0.0204	1.236	Additive
FFN	0.0675	0.1481	0.455	Non-additive
NVB	0.0000	0.1928	0.0	Non-additive
NFB	1.6548	1.6024	1.033	Additive
PH	49.3517	52.0557	0.948	Non-additive

σ²GCA and σ²SCA are variance-component estimates, with negative raw values set to zero before computing the ratio. “Undefined” indicates σ²SCA = 0. MIC = micronaire; FL = fiber length; UI = uniformity index; FS = fiber strength; FFN = first fruiting node; NVB = number of vegetative branches; NFB = number of fruiting branches; PH = plant height.

### Error variance, precision, and broad-sense heritability

Coefficients of variation from the combined error term (Table 8) ranged from 0.13% for UI to 25.81% for NVB, indicating good experimental precision for the fiber-quality traits and comparatively higher plot-to-plot variation for NVB. The genotype-to-error mean square ratio exceeded 1 for every trait, confirming that genetic differences among genotypes were large relative to residual error. Broad-sense heritability, estimated on a combined-environment basis, ranged from 0.745 for NVB to 0.996 for FL, indicating that most of the phenotypic variation among genotypes was genetic in origin, with a larger environmental/interaction component for NVB.

**Table 8:** Error mean square, genotype-to-error ratio, coefficient of variation, and broad-sense heritability for eight fiber-quality and agronomic traits, combined analysis across normal-irrigation and drought environments.

Trait	Error MS	Genotype MS	Genotype/Error	CV (%)	Broad-sense H²
MIC	0.0140	0.7532	53.77	3.03	0.981
FL	0.0171	4.5360	264.62	0.38	0.996
UI	0.0127	1.0844	85.52	0.13	0.988
FS	0.0107	0.3915	36.56	0.99	0.973
FFN	0.3433	2.7512	8.01	7.77	0.875
NVB	0.4030	1.5774	3.91	25.81	0.745
NFB	2.9331	24.9960	8.52	7.40	0.883
PH	44.4871	715.0460	16.07	3.67	0.938

CV = coefficient of variation based on the combined error mean square and overall trait mean. Broad-sense H² = σ²G / (σ²G + MSE/re), where σ²G = (MS genotype − MSE)/(re). MIC = micronaire; FL = fiber length; UI = uniformity index; FS = fiber strength; FFN = first fruiting node; NVB = number of vegetative branches; NFB = number of fruiting branches; PH = plant height.

### Heterosis analysis

#### Mid-parent heterosis under normal conditions

The mid-parent heterosis (HMP) values for all 21 crosses under normal irrigation are shown in Table 9. For micronaire, 12 crosses (57.1%) exhibited positive heterosis, ranging from − 2.5% to 10.67%, with Giza 96 × Giza 45 showing the highest (10.67%) and Giza 93 × Giza 45 the lowest (− 3.45%). For fiber length, 12 crosses (57.1%) showed positive heterosis, ranging from − 0.34% to 1.16%, with Giza 96 × Giza 45 reaching the maximum (1.16%). The uniformity index followed a similar trend, with 12 positive crosses and a peak of 0.46% for Giza 96 × Giza 45 (*P* < 0.001). Fiber strength showed the broadest positive response, with 13 crosses (61.9%) exhibiting positive heterosis, reaching a maximum of 0.46% in Giza 96 × Giza 45. Among agronomic traits, the first fruiting node had the highest proportion of positive heterosis crosses (76.2%, 16 of 21), with a notable maximum of 44.00% for Giza 87 × Giza 45, indicating substantially greater fruiting branch development than the mid-parent value. The number of vegetative branches showed positive heterosis in 33.3% of crosses, with Giza 87 × Giza 94 showing 100.00% heterosis, though the desirability of this trait varies with breeding goals. The number of fruiting branches had 71.4% positive crosses, with G.89×G.86 × Giza 45 achieving the highest value at 56.04%. Plant height heterosis ranged from − 20.27% to 20.83%, with Giza 87 × Giza 45 recording the maximum (20.83%).

**Table 9:** Mid-parent heterosis (%) under normal conditions.

Cross	MIC (FF)	FL (mm)	UI (%)	FS (g/tex)	FFN	NVB	NFB	PH (cm)
Giza 45 ×								
Giza 70 × Giza 45	2.16	0.21	0.09	0.69	10.34	45.45*	7.87	9.43***
Giza 85 × Giza 45	-0.63	-0.07	-0.03	-0.22	30.77***	0.00	15.56*	12.58***
Giza 87 × Giza 45	1.81	0.15	0.06	0.49	44.00***	50.00	40.23***	20.83***
Giza 92 × Giza 45	1.77	0.18	0.07	0.59	35.71***	40.00	15.22*	-4.46*
Giza 93 × Giza 45	6.13*	0.56**	0.23*	1.90***	-3.45	40.00	-10.64	-10.97***
Giza 96 × Giza 45	10.67***	1.16***	0.46***	0.68	15.38*	-42.86**	40.23***	-0.65
G.89×G.86 × Giza 45	-2.49	-0.28	-0.11	-0.89	18.52**	20.00	56.04***	9.55***
***Giza 94 ×***								
Giza 70 × Giza 94	1.59	0.19	0.08	0.64	-3.45	-45.45*	8.33	1.28
Giza 85 × Giza 94	1.15	0.15	0.06	0.51	15.38*	-40.00	-21.65***	-20.27***
Giza 87 × Giza 94	1.31	0.14	0.06	0.46	20.00**	100.00***	25.53***	20.57***
Giza 92 × Giza 94	4.72*	0.10	0.04	0.33	28.57***	0.00	5.05	0.00
Giza 93 × Giza 94	5.38*	0.61**	0.24**	2.12***	3.45	0.00	-10.89	-1.32
Giza 96 × Giza 94	-1.46	-0.19	-0.08	-0.66	15.38*	-14.29	14.89*	13.16***
G.89×G.86 × Giza 94	-2.50	-0.34	-0.16	-1.11*	11.11	0.00	16.33**	14.29***
***Giza 86 ×***								
Giza 70 × Giza 86	0.35	0.04	0.02	0.29	0.00	-53.85**	4.26	5.00**
Giza 85 × Giza 86	-0.34	-0.04	-0.02	0.02	24.14***	0.00	-9.47	-19.74***
Giza 87 × Giza 86	-1.07	-0.12	-0.05	-0.21	21.43**	-40.00	15.22*	15.86***
Giza 92 × Giza 86	-0.58	-0.12	-0.03	-0.08	-9.68	-33.33	-5.15	-6.33**
Giza 93 × Giza 86	3.26	0.38	0.15	1.40**	0.00	16.67	5.05	-2.56
Giza 96 × Giza 86	-1.33	-0.18	-0.07	-0.42	17.24**	-12.50	-15.22*	-14.10***
G.89×G.86 × Giza 86	-1.77	-0.24	-0.09	-0.60	6.67	-66.67***	22.92***	3.80*
**LSD₀.₀₅**	**0.20**	**0.17**	**0.17**	**0.12**	**1.01**	**1.17**	**3.24**	**8.42**

* *P* ≤ 0.05; ** *P* ≤ 0.01; *** *P* ≤ 0.001.MIC = Micronaire (FF); FL = Fiber Length (UHML mm); UI% = Uniformity Index; FS = Fiber Strength (g/tex); FFN = First Fruiting Node; NVB = No. Vegetative Branches; NFB = No. Fruiting Branches; PH = Plant Height (cm). GCA = General Combining Ability; SCA = Specific Combining Ability. * *P* ≤ 0.05; ** *P* ≤ 0.01; *** *P* ≤ 0.001.

#### Mid-parent heterosis under drought conditions

Under drought stress (Table 10), heterosis patterns changed considerably for several traits. For micronaire, the proportion of positively heterotic crosses declined to 42.9% (9 crosses), though the maximum value increased dramatically to 35.49% for Giza 96 × Giza 45, more than three times the normal-condition maximum. Fiber length showed fewer positive heterotic crosses under drought (38.1%, 8 crosses) compared to normal conditions, but the maximum increased to 8.48% for Giza 85 × Giza 86. Uniformity index maintained 52.4% positive crosses, with a maximum of 2.01% for Giza 85 × Giza 86, and fiber strength improved to 66.7% positive crosses under drought, with a maximum of 6.57% for Giza 85 × Giza 86, identifying this cross as the best overall fiber-quality combiner under stress conditions. For agronomic traits under drought, the proportion of positively heterotic crosses declined for the number of fruiting branches (from 71.4% to 42.9%) and the number of vegetative branches (from 33.3% to 42.9% with a maximum of 50.00% for G.89×G.86 × Giza 94, *P* ≤ 0.05). The first fruiting node remained highly heterotic under drought, with 66.7% positive crosses and a best value of 33.33% for Giza 87 × Giza 94. Plant height showed equal proportions of positive crosses under both conditions (52.4%), with a maximum of 11.76% for Giza 70 × Giza 45 (*P* ≤ 0.01) under drought.

**Table 10:** Mid-parent heterosis (%) under drought conditions.

Cross	MIC (FF)	FL (mm)	UI (%)	FS (g/tex)	FFN	NVB	NFB	PH (cm)
Giza 45 ×								
Giza 70 × Giza 45	6.31*	-1.41***	0.45***	2.29**	13.21*	-40.00*	7.53	11.76**
Giza 85 × Giza 45	-7.43***	-3.02***	0.36***	-1.75*	1.96	-25.00	-9.76	-12.76**
Giza 87 × Giza 45	-9.80***	-2.93***	0.39***	-1.60*	25.49***	25.00	27.50***	5.97
Giza 92 × Giza 45	0.33	-0.81*	-0.33***	1.52*	24.14***	-20.00	22.08**	7.35*
Giza 93 × Giza 45	11.37***	2.08***	1.20***	4.75***	0.00	25.00	2.33	2.26
Giza 96 × Giza 45	35.49***	-6.88***	-0.43***	-6.27***	14.29**	25.00	-7.14	1.98
G.89×G.86 × Giza 45	0.65	-1.49***	-0.66***	2.18**	-6.67	33.33	-14.61*	9.52*
***Giza 94 ×***								
Giza 70 × Giza 94	-2.43	-3.43***	-0.95***	-4.56***	-1.89	-33.33*	-22.11***	2.16
Giza 85 × Giza 94	-24.50***	0.41	-0.46***	0.45	1.96	-40.00*	-7.14	9.23*
Giza 87 × Giza 94	-4.37*	2.36***	0.60***	3.02***	33.33***	0.00	-2.44	-1.90
Giza 92 × Giza 94	8.69***	-2.80***	-0.63***	-3.30***	-3.45	-16.67	3.80	-5.04
Giza 93 × Giza 94	-2.23	-4.94***	-1.09***	0.05	7.14	0.00	4.55	7.35*
Giza 96 × Giza 94	-4.38*	-5.29***	-1.50***	-2.35**	14.29**	-60.00***	13.95*	5.65
G.89×G.86 × Giza 94	-3.83*	-2.03***	-0.88***	0.34	0.00	50.00*	-12.09*	3.88
***Giza 86 ×***								
Giza 70 × Giza 86	-21.17***	5.75***	0.31***	4.51***	-1.89	-45.45**	-22.11***	-7.59*
Giza 85 × Giza 86	-8.63***	8.48***	2.01***	6.57***	1.96	-11.11	-2.38	-7.35*
Giza 87 × Giza 86	2.47	8.29***	1.53***	1.02	33.33***	11.11	12.20	-9.09*
Giza 92 × Giza 86	2.49	8.27***	0.81***	3.33***	10.34*	27.27	8.86	-4.83
Giza 93 × Giza 86	-8.00***	3.54***	0.46***	6.50***	7.14	-33.33	-6.82	-4.23
Giza 96 × Giza 86	19.85***	-0.28	0.46***	-2.96***	14.29**	11.11	0.00	-4.56
G.89×G.86 × Giza 86	-4.32*	-1.02**	-0.91***	0.15	-6.67	42.86	-9.89	-9.63*
**LSD₀.₀₅**	**0.17**	**0.26**	**0.17**	**0.17**	**0.86**	**0.95**	**3.07**	**14.15**

* *P* ≤ 0.05; ** *P* ≤ 0.01; *** *P* ≤ 0.001.MIC = Micronaire (FF); FL = Fiber Length (UHML mm); UI% = Uniformity Index; FS = Fiber Strength (g/tex); FFN = First Fruiting Node; NVB = No. Vegetative Branches; NFB = No. Fruiting Branches; PH = Plant Height (cm). GCA = General Combining Ability; SCA = Specific Combining Ability. * *P* ≤ 0.05; ** *P* ≤ 0.01; *** *P* ≤ 0.001.

#### Better-parent heterosis under normal and drought conditions

Better-parent heterosis (HBP) estimates under normal irrigation (Table 11) were generally lower in magnitude than mid-parent values but remained significant for several crosses. For fiber quality under normal conditions, Giza 87 × Giza 45 displayed notable positive HBP for the first fruiting node (38.46%) and number of fruiting branches (38.64%), confirming that this cross genuinely exceeded both parents for these productivity-related traits. The cross Giza 96 × Giza 45 showed the best HBP for micronaire (− 6.94%, *P* ≤ 0.01), a negative value being desirable for this trait as well as for fiber length (− 1.61%, *P* ≤ 0.01), both indicating superiority over the better parent. G.89×G.86 × Giza 45 exhibited the highest positive HBP for the number of fruiting branches (47.92%) under normal conditions. The cross Giza 93 × Giza 45 showed the most negative HBP for plant height (− 11.54%, *P* ≤ 0.01), suggesting appreciable hybrid vigor for compact stature. Under drought stress (Table 12), several crosses exhibited genuine transgressive heterosis, exceeding that of the better parent for yield-related components. Giza 96 × Giza 45 maintained the best positive HBP for micronaire under drought (21.27%). For fiber length under drought, Giza 85 × Giza 86 showed positive HBP (2.47%, *P* ≤ 0.01). In terms of agronomic performance, Giza 87 × Giza 45 showed positive HBP for the first fruiting node (23.08%) and the number of fruiting branches (18.60%) under drought, maintaining its performance advantage over parental values even under stress. Giza 92 × Giza 45 also exhibited significant positive HBP for the number of fruiting branches (9.30%, *P* ≤ 0.05) under drought. Conversely, several crosses showed increasingly negative HBP for fiber traits under drought stress, reflecting the general challenge of maintaining fiber quality in hybrids under water-limited conditions. The cross Giza 70 × Giza 86 exhibited the most negative HBP for micronaire under drought (− 35.86%), indicating substantial negative heterosis for this quality parameter under stress.

**Table 11:** Better-parent heterosis (%) under normal conditions.

Cross	MIC (FF)	FL (mm)	UI (%)	FS (g/tex)	FFN	NVB	NFB	PH (cm)
Giza 45 ×								
Giza 70 × Giza 45	-9.93***	-0.13	0.00	-0.45	-5.88	33.33	4.35	7.41***
Giza 85 × Giza 45	-14.69***	-3.39***	-0.88***	-4.71***	21.43**	0.00	10.64	8.97***
Giza 87 × Giza 45	0.33	-0.11	-0.73***	-0.89	38.46***	20.00	38.64***	11.54***
Giza 92 × Giza 45	-10.90***	-1.75***	-0.37***	-2.06***	18.75**	40.00	8.16	-5.06*
Giza 93 × Giza 45	-0.29	0.46	-0.10	-1.25*	-17.65**	40.00	-17.65**	-11.54***
Giza 96 × Giza 45	-6.94**	-1.61***	0.08	-3.74***	7.14	-55.56***	38.64***	-1.28
G.89×G.86 × Giza 45	-18.39***	-5.44***	-0.80***	-3.19***	6.67	20.00	47.92***	8.86***
***Giza 94 ×***								
Giza 70 × Giza 94	-2.36	-1.86***	-0.53***	-6.35***	-17.65**	-50.00*	4.00	-2.47
Giza 85 × Giza 94	0.23	-0.85**	-0.10	-1.10	7.14	-40.00	-24.00***	-21.33***
Giza 87 × Giza 94	-14.72***	-1.99***	-0.05	-6.73***	15.38	60.00*	18.00**	13.33***
Giza 92 × Giza 94	1.45	-0.38	-0.21*	-3.17***	12.50	0.00	4.00	-2.53
Giza 93 × Giza 94	-5.11*	-1.69***	-0.12	-1.00	-11.76	0.00	-11.76	-2.60
Giza 96 × Giza 94	-3.03	-0.57*	-0.39***	-2.36***	7.14	-33.33*	8.00	11.69***
G.89×G.86 × Giza 94	-4.60*	-3.26***	-0.17	-4.87***	0.00	0.00	14.00*	11.39***
***Giza 86 ×***								
Giza 70 × Giza 86	-4.75*	-3.39***	-0.64***	-2.29***	-5.88	-57.14**	2.08	3.70
Giza 85 × Giza 86	-2.53	-0.50	-0.13	-3.10***	20.00**	-14.29	-10.42	-22.78***
Giza 87 × Giza 86	-17.61***	-3.61***	-0.10	-3.01***	13.33	-57.14**	10.42	6.33**
Giza 92 × Giza 86	-4.90*	-2.02***	-0.32**	-1.28*	-12.50	-42.86*	-6.12	-6.33**
Giza 93 × Giza 86	-8.10***	-3.29***	-0.26*	-0.28	-5.88	0.00	1.96	-3.80
Giza 96 × Giza 86	-1.62	-1.25***	-0.43***	-3.41***	13.33	-22.22	-18.75**	-15.19***
G.89×G.86 × Giza 86	-2.63	-1.78***	-0.14	-1.47*	6.67	-71.43***	22.92**	3.80
**LSD₀.₀₅**	**0.20**	**0.17**	**0.17**	**0.12**	**1.01**	**1.17**	**3.24**	**8.42**

* *P* ≤ 0.05; ** *P* ≤ 0.01; *** *P* ≤ 0.001.

**Table 12:** Better-parent heterosis (%) under drought conditions.

Cross	MIC (FF)	FL (mm)	UI (%)	FS (g/tex)	FFN	NVB	NFB	PH (cm)
Giza 45 ×								
Giza 70 × Giza 45	-5.00	-2.53***	0.00	1.92*	11.11	-57.14***	0.00	8.57*
Giza 85 × Giza 45	-16.86***	-4.12***	0.03	-2.53**	0.00	-40.00*	-13.95	-16.06***
Giza 87 × Giza 45	-17.37***	-7.76***	-0.26*	-2.60**	23.08**	0.00	18.60*	4.41
Giza 92 × Giza 45	-2.85	-2.40***	-0.69***	-0.03	12.50*	-42.86**	9.30	4.29
Giza 93 × Giza 45	0.88	-1.16**	1.07***	1.21	-6.67	0.00	2.33	1.49
Giza 96 × Giza 45	21.27***	-8.18***	-0.78***	-7.93***	6.67	0.00	-9.30	1.52
G.89×G.86 × Giza 45	-9.85***	-4.61***	-0.83***	0.22	-17.65**	33.33	-17.39*	4.55
***Giza 94 ×***								
Giza 70 × Giza 94	-20.94***	-4.66***	-1.31***	-5.16***	-3.70	-42.86**	-26.00***	1.43
Giza 85 × Giza 94	-24.88***	-0.89*	-0.94***	0.25	0.00	-40.00*	-13.33	2.90
Giza 87 × Giza 94	-6.88**	-2.87***	-0.85***	0.96	30.77***	0.00	-11.11	-2.61
Giza 92 × Giza 94	0.08	-4.51***	-1.08***	-3.82***	-12.50*	-28.57*	-8.89	-5.71
Giza 93 × Giza 94	-3.63	-8.10***	-2.00***	-4.26***	0.00	0.00	2.22	5.80
Giza 96 × Giza 94	-22.41***	-6.76***	-2.64***	-3.12***	6.67	-60.00**	8.89	2.90
G.89×G.86 × Giza 94	-4.02*	-5.27***	-1.51***	-2.55**	-11.76*	20.00	-13.04	-2.90
***Giza 86 ×***								
Giza 70 × Giza 86	-35.86***	-0.11	-0.73***	1.99*	-3.70	-57.14***	-26.00***	-10.67**
Giza 85 × Giza 86	-8.67***	2.47***	1.07***	3.56***	0.00	-20.00	-8.89	-16.00***
Giza 87 × Giza 86	0.31	6.40***	1.48***	-0.06	30.77***	0.00	2.22	-13.33***
Giza 92 × Giza 86	-5.16*	2.74***	-0.15	-0.31	0.00	0.00	-4.44	-8.00*
Giza 93 × Giza 86	-8.83***	-0.20	-0.02	5.03***	0.00	-40.00*	-8.89	-9.33*
Giza 96 × Giza 86	-2.34	-5.57***	0.21*	-6.61***	6.67	0.00	-4.44	-10.67**
G.89×G.86 × Giza 86	-4.65*	-4.59***	-1.67***	0.00	-17.65**	25.00	-10.87	-18.67***
**LSD₀.₀₅**	**0.17**	**0.26**	**0.17**	**0.17**	**0.86**	**0.95**	**3.07**	**14.15**

* *P* ≤ 0.05; ** *P* ≤ 0.01; *** *P* ≤ 0.001.

## Discussion

### Drought reshapes the genetic architecture of fiber quality traits

The most consequential finding of the present study is the systematic shift in gene action for fiber quality traits from predominantly additive under normal irrigation to predominantly non-additive under drought stress. Under well-watered conditions, the GCA/SCA ratios for micronaire (13.0), fiber length (81.6), and uniformity index (19.2) indicate that additive allelic effects account for the large majority of genetic variance in these traits a pattern consistent with previous Egyptian cotton studies^3,15,21,27^ and with the expectation that traits subject to long-term directional selection accumulate predominantly additive genetic variance^28^. The corresponding high narrow-sense heritability’s (0.87–0.98) confirm that selection for fiber quality under favorable water supply would be efficient in early generations, consistent with the success of pedigree breeding methods in Egyptian cotton improvement programs.

Under drought, all fiber quality traits showed GCA/SCA ratios below unity and sharply reduced narrow-sense heritability, while broad-sense heritability remained high. This patternhigh H²b with low h²nindicates that drought selectively suppresses the additive component while amplifying dominance and epistatic variance. Mechanistically, this shift may reflect several processes. Fiber elongation depends on sustained turgor pressure to drive cell expansion during the primary wall synthesis phase (approximately 0–20 days post-anthesis), a process that is directly disrupted by drought-induced reduction in water potential^4^. The sensitivity of turgor-dependent elongation to water availability may create opportunities for dominance and epistatic buffering in heterozygous genotypes, particularly through complementarity in drought-adaptive mechanisms such as osmotic adjustment capacity, root architecture, or stomatal conductance regulation. Secondary wall deposition and cellulose accumulation, which determine fiber strength and contribute to micronaire, are regulated by carbon allocation and sucrose transport into developing fibers processes sensitive to the photosynthate supply reduction that accompanies drought-induced stomatal closure^5^. The stress-dependent expression of these carbon partitioning pathways may reveal dominance relationships between alleles regulating carbon transport under low versus adequate photosynthate supply.

Similar shifts from additive to non-additive gene action under environmental stress have been documented in other cotton systems^29,30^ and are consistent with the general principle in quantitative genetics that non-additive genetic effects may be more fully expressed when alleles are challenged by stressful conditions^31^. The practical implication is that breeding strategies optimized for fiber quality improvement under normal conditions, particularly early-generation selection, may be substantially less effective under drought, and that programs targeting drought-affected environments should be designed and evaluated separately.

### Environmental regulation of additive and non-additive gene effects

The contraction of tester GCA significance under drought, from all fiber traits under normal conditions to only micronaire and plant height under stress, indicates that the genetic contributions of testers are more environmentally sensitive than those of lines. This asymmetry may reflect differences in the breadth and stability of the alleles contributed by lines versus testers: lines typically represent a wider range of genetic backgrounds in L × T designs, and their allelic effects may be more robust to environmental perturbation because they sample more of the allelic diversity relevant to the trait. In contrast, tester contributions to specific combining ability were amplified under drought conditions, as evidenced by highly significant SCA interactions for all traits, including previously non-significant fiber strength. This pattern suggests that drought creates conditions in which the complementarity or suppression between specific line-tester allele combinations becomes more influential than the main effects of either parent, consistent with a model in which stress reveals conditional or context-dependent gene interactions that are buffered under favorable conditions^2,32,33^.

The emergence of Giza 85 as the superior fiber-length combiner under drought (GCA = 1.08)despite moderate or negative fiber-quality GCA under normal conditions exemplifies genotype-specific drought adaptation at the combining ability level. This shift could reflect the activation of drought-responsive genetic pathways in Giza 85 that contribute positively to fiber length only under water deficit, or the release from competitive suppression between vegetative growth and fiber development that may occur when drought limits overall biomass accumulation. Abdelghany et al. ^2^ similarly documented environment-dependent GCA shifts in Egyptian cotton and cautioned against generalizing combining ability estimates across irrigation regimes.

The stability of Giza 92’s positive fiber-quality GCA across both environments (significant for all four fiber traits in both) may indicate that its complex pedigree Giza 84 × (Giza 74 × Giza 68) confers broad-spectrum allelic contributions to fiber quality that are less dependent on water availability. This genotype may harbor alleles that support fiber development through mechanisms that remain effective under moderate water deficit, such as constitutively elevated sucrose transport capacity or robust turgor maintenance mechanisms.

The broader pattern observed here a decline in the relative contribution of additive variance for fiber traits under water deficit is consistent with findings outside Egyptian cotton germplasm. In a full-diallel study of upland cotton (*G. hirsutum*) evaluated under well-watered and deficit irrigation, Jinks-Hayman analysis found dominance effects to be more important than additive effects for all measured traits under both regimes, with the additive-dominance imbalance more pronounced under water deficit^41^. Together with the present results, this suggests that a stress-associated shift toward non-additive gene action for fiber traits may not be specific to a single genetic background or cotton species, though the mating designs, stress protocols, and trait sets differ enough between studies that this comparison should be treated as suggestive rather than a formal test of generality; multi-species, multi-environment trials using a common design would be needed to establish how widely this pattern holds.

### Genetic control of plant architecture and reproductive development under drought

Plant height and fruiting branch number were predominantly non-additively controlled under both environments, consistent with their complex polygenic architecture and known environmental sensitivity^6,34^. Plant height in cotton is regulated by multiple hormonal pathwaysauxin promotes internode elongation, gibberellins stimulate stem growth, and ABA, which accumulates under drought, suppresses vegetative elongation while redirecting resources toward reproductive development^24^. The predominantly non-additive gene action for plant height under both environments suggests that these hormonal regulatory interactions create epistatic networks in which the phenotypic outcome depends on specific allele combinations rather than additive dosage effects. Under drought, the further reduction in plant height GCA/SCA ratio (from 0.29 to 0.10) is consistent with increased ABA-mediated suppression of additive growth contributions and greater dependence on specific epistatic buffering mechanisms. The first fruiting node stands apart from other agronomic traits in showing an additive-dominant GCA/SCA ratio (1.11) and substantially increased narrow-sense heritability (0.43) under drought. The first fruiting node position reflects the number of main-stem nodes below the first sympodial branch, a developmental switch from vegetative to reproductive growth that is sensitive to photoperiod, temperature, and plant stress status. Under water deficit, the transition from vegetative to reproductive growth may be partially accelerated through ABA and ethylene signaling, with the additive genetic component of this response becoming more detectable because stress conditions reduce the environmental variance that masks additive allelic differences under favorable growth conditions. This interpretation is consistent with the observation that Giza 87 contributed the most positive GCA for the first fruiting node under drought (0.88), suggesting that this genotype carries alleles that promote earlier reproductive transition under stress, a potentially adaptive response to water limitation.

## Specific combining ability

The significant SCA effects observed across several crosses, particularly for agronomic traits in both environments, indicate favorable non-additive gene interactions beyond what would be predicted from parental GCA values alone. Under normal conditions, the strong positive SCA of Giza 96 × Giza 45 for micronaire, fiber length, and uniformity index (all *P* ≤ 0.01) is particularly noteworthy, especially given that Giza 96 has unfavorable GCA for fiber length. This outcome suggests that complementary epistatic interactions between Giza 96 and Giza 45 overcome the individual parental deficiencies, a phenomenon consistent with the concept of specific combining ability as described by Kempthorne^24^, who established that SCA effects reflect deviations from additive expectations due to non-additive gene interactions. Al-Hibbiny^31^ similarly observed crosses in which favorable SCA effects arose from parents with moderate or negative GCA, and concluded that SCA analysis provides information that is complementary to, and not always predictable from, GCA alone.

The large positive SCA effects for agronomic traits, notably Giza 87 × Giza 45 for the first fruiting node and number of fruiting branches, and Giza 93 × Giza 86 for number of fruiting branches and plant height under normal conditions, indicate specific epistatic interactions that enhance plant productivity potential in these hybrid combinations. The negative SCA for the number of fruiting branches in Giza 93 × Giza 45 (− 4.024, *P* ≤ 0.01) under normal conditions, conversely, suggests suppressive interactions unfavorable for yield potential, despite both parents having individually acceptable GCA profiles. Abdel-Aty et al. ^21^ documented similar negative SCA interactions in Egyptian cotton crosses and cautioned that parental GCA rankings are not reliable predictors of all cross-specific hybrid performance outcomes.

Under drought stress, the restructuring of SCA patterns in Giza 93 × Giza 45, with Giza 93 now showing the highest positive SCA for fiber length (1.427) and uniformity index (0.777), demonstrates the highly environment-dependent nature of non-additive gene interactions in Egyptian cotton. This aligns with the genotype-by-environment interaction framework described by Lamlom et al. ^3^, who showed that specific cross combinations may exhibit superior performance under stress conditions while performing poorly under favorable conditions, and vice versa. The large positive SCA of Giza 85 × Giza 94 for plant height under drought (15.738, *P* ≤ 0.01) in the absence of significant effects for yield-related traits suggests that plant structure adaptation to drought may be governed by specific gene interactions distinct from those controlling fiber quality and productivity. EL-Shazly et al.^35^ observed similar environment-specific SCA patterns for plant architecture traits in Egyptian cotton under stress conditions.

### Biological significance of heterosis under water deficit

The amplification of fiber quality heterosis under drought, most strikingly in Giza 85 × Giza 86which simultaneously showed 8.48% HMP for fiber length, 2.01% for uniformity index, and 6.57% for fiber strength under drought, compared to near-zero HMP for these traits under normal conditions, suggests that specific heterozygous combinations can substantially buffer fiber development against water deficit. This “stress heterosis” likely reflects complementary genetic effects in which favorable alleles from each parent contribute to different components of drought adaptation: one parent may contribute superior osmotic adjustment capacity while the other contributes more effective carbon partitioning toward fiber development, or one may contribute earlier stomatal closure to conserve water while the other contributes more efficient cellulose synthesis under reduced carbon supply.

The better-parent heterosis results for Giza 85 × Giza 86 (HBP = 2.47% for fiber length; 3.56% for fiber strength under drought) confirm that this cross genuinely exceeded the superior parent for these traits, meeting the classical definition of heterobeltiosis. While the absolute percentage values appear modest, their practical significance in the context of extra-long staple cotton markets is considerable, given that market classification thresholds for premium grades are narrow and small improvements in fiber length or strength may shift classification to a higher market grade^30,36,37^. The consistent finding of higher fiber quality heterosis under drought than under normal conditions across multiple crosses supports a model in which heterozygous buffering of fiber quality is more important and more expressed under stress, consistent with Falconer^28^ observation that dominance effects tend to become more expressed as genetic buffering mechanisms are challenged by environmental stress.

### Implications for climate-resilient cotton adaptation

The results collectively suggest a framework for environment-specific cotton breeding. Under normal irrigation, the high narrow-sense heritability and additive gene action for fiber quality support early-generation pedigree or bulk selection, with Giza 45 (tester), Giza 70, and Giza 87 (lines) as priority parents. The high additive control documented here, consistent with prior Egyptian cotton studies, suggests that genetic gains from selection should be predictable and relatively stable across environments with adequate water^2,21,31,37^. Under drought conditions, the dominance of non-additive gene action for fiber quality and the reduced narrow-sense heritability indicate that hybridization approaches that exploit SCA effects are likely to be more productive than selection-based methods. Giza 85 × Giza 86 is the most promising identified cross for simultaneous fiber quality maintenance under drought; Giza 93 × Giza 45 showed favorable SCA for fiber length and uniformity index under drought. Giza 85 and Giza 92 are recommended as priority parental lines for drought-targeted crossing programs. Giza 45 retains value as a drought-environment tester for GCA screening despite its reduced tester-GCA significance under stress, given its broad combining ability profile. The environment-specific GCA rankings documented here particularly the emergence of Giza 85 as a superior drought combiner despite moderate performance under normal conditions underscore the necessity of evaluating parental combining ability under target stress conditions rather than extrapolating from normal-condition estimates. Maintaining separate crossing programs and parental evaluations for each target environment, as advocated in the genotype-by-environment interaction framework for Egyptian cotton^38–40^, appears warranted by these data.

Translating a hybridization-based strategy for drought environments into practice depends on the feasibility of producing hybrid seed at a commercial scale. Egyptian cotton is predominantly self-pollinated, and the F^1^ hybrids evaluated in this study were produced by hand emasculation and pollination, as is standard for experimental line × tester populations. Scaling this approach to commercial hybrid seed production would require either continued manual emasculation and pollination, labor-intensive and costly at the field scale, or the development and deployment of a cytoplasmic male-sterility/restoration system, which has not yet been established as a routine tool in Egyptian cotton breeding programs^42^. Whether a hybridization strategy is economically justified for drought-prone areas of the Nile Delta would therefore depend on weighing the fiber-quality and yield gains from exploiting SCA against the added cost of hybrid seed production, and on parallel investment in seed multiplication infrastructure; we present this as a consideration for future applied breeding and seed-system research rather than a claim that commercial hybrid cotton production is already feasible at scale in this region.

The finding that both additive and non-additive gene action contribute to fiber quality and yield-related traits under a single environment is broadly consistent with prior Egyptian cotton combining-ability studies, including Abdelghany et al.^2^ and Abdel-Aty et al.^21^, which reported significant GCA and SCA effects for yield and fiber traits under single-environment field trials at the same station but did not compare gene action between contrasting water regimes. The present study extends this line of work by evaluating the same parents and crosses under both normal and drought conditions, allowing a direct within-trial comparison that these earlier studies could not make. The shift toward relatively greater non-additive variance under stress reported here also parallels findings from Mahdy et al.^8^, who found GCA/SCA ratios below one for cotton yield and quality traits under salinity stress, suggesting that a relative shift toward non-additive gene action under abiotic stress may not be specific to drought and could reflect a more general feature of stress response in this species; confirming this would require comparison across multiple stress types within a single genetic background, which is beyond the scope of the present study.

### Future directions: integrating quantitative genetics and molecular biology

Several key questions remain to be addressed in future research. The molecular basis of the observed GCA shifts under drought, particularly the emergence of Giza 85 as a superior drought combiner, warrants investigation through transcriptomic and metabolomic profiling of fiber development under normal and drought conditions in key parental genotypes and their crosses. Quantitative trait locus (QTL) mapping or genome-wide association studies under contrasting irrigation regimes may identify loci whose effects are environment-dependent, providing targets for marker-assisted selection in drought breeding programs. The observation that non-additive gene effects for fiber quality are amplified under drought raises the possibility that stress-responsive epigenetic mechanisms, including DNA methylation changes and small RNA pathways that regulate gene expression in heterozygous backgrounds, may contribute to the observed dominance and epistasis patterns. This hypothesis could be tested using methylation-sensitive genomic approaches. Multi-environment and multi-year validation of the promising cross combinations identified here, particularly Giza 85 × Giza 86 and Giza 93 × Giza 45 under drought conditions, is essential before they can be advanced in commercial hybrid development programs.

Several aspects of the experimental design warrant caution in interpreting and generalizing these results. First, data were collected from a single growing season (2025) at a single location (Sakha Agricultural Experimental Research Station); genotype × environment interactions for drought response are well documented in cotton, and year-to-year variation in temperature, rainfall timing, and stress severity could affect GCA and SCA estimates, and consequently the GCA/SCA ratios and heritability estimates reported here. Second, the parental set (7 lines × 3 testers) provides a comparatively limited sampling of the genetic diversity available in Egyptian cotton, and the specific combining-ability patterns and gene-action classifications identified may not extend to other genetic backgrounds. For these reasons, the findings reported here should be regarded as evidence from a single environment and genetic sample, and generalization to other seasons, locations, or germplasm should be made with appropriate caution pending multi-environment validation. Third, drought stress was imposed by restricting irrigation to three applications rather than by direct control of soil water content, and soil moisture at the start and end of the stress period and a physiological indicator of water deficit (e.g., leaf relative water content or midday leaf water potential) were not measured; consequently, the degree of water deficit actually experienced by the plants is inferred from the irrigation regime and prevailing temperature rather than confirmed directly.

## Conclusions

This study shows drought stress reshapes Egyptian cotton’s genetic structure for fiber quality and development traits. Under normal water conditions, traits such as fiber length, uniformity, micronaire, and strength are mainly controlled by additive effects with high heritability, making early selection effective. Drought significantly alters this, shifting control to non-additive effects, with GCA/SCA ratios dropping and heritability estimates changing. The suppression of additive effects suggests drought triggers genetic buffering, possibly via stress-adaptive pathways in heterozygous plants. For agronomic traits, the first fruiting node’s genetic control becomes environment-dependent under drought, which could aid breeding for drought-tolerant phenology. GCA rankings shift between environments: Giza 85 excels under drought in fiber length, while Giza 92 maintains consistent GCA across traits. Crosses such as Giza 85 × Giza 86 show heterosis under water stress, indicating that gene interactions buffer fiber development. Breeding strategies should be environment-specific: pedigree or bulk selection with specific Giza lines under normal conditions, and hybridization that exploits non-additive variance for drought. Key crosses include Giza 85 × Giza 86 and Giza 85 × Giza 92. The environment-dependent genetic control emphasizes the importance of evaluating combining ability, heritability, and heterosis under stress conditions and advocates for separate breeding populations for each irrigation regime. Future work combining genetics, transcriptomics, and molecular markers will be crucial for understanding drought effects and developing climate-resilient Egyptian cotton.

## Supplementary Information

Below is the link to the electronic supplementary material.

Supplementary Material 1

## Data Availability

The datasets used and/or analyzed during the current study available from the corresponding author (Sobhi F. Lamlom) on reasonable request.

## References

[1] Abd, E. L. A. Evaluation of genetic gains of some quantitative characters in Egyptian cotton cross (Giza 86× menoufi) under water deficit stress. *Sci. Rep.* **12**, 15227 (2022). 36075945 10.1038/s41598-022-18966-3 PMC9458738

[2] Abdelghany, A. M. et al. Combining ability of Egyptian cotton (Gossypium barbadense L.) reveals genetic potential for improved yield and fiber quality. *J. Cotton Res.* **7**, 12 (2024).

[3] Lamlom, S. F. et al. Genetic improvement of Egyptian cotton (Gossypium barbadense L.) for high yield and fiber quality properties under semi arid conditions. *Sci. Rep.* **14**, 7723 (2024). 38565894 10.1038/s41598-024-57676-w PMC10987534

[4] Balci, S., Cinar, V. M. & Unay, A. Estimating gene action and combining ability for yield and fiber quality in cotton (Gossypium hirsutum L). *Genetika* **55**, 61–69 (2023).

[5] Campbell, B. & Jones, M. Assessment of genotype× environment interactions for yield and fiber quality in cotton performance trials. *Euphytica* **144**, 69–78 (2005).

[6] Ul-Allah, S., Rehman, A., Hussain, M. & Farooq, M. Fiber yield and quality in cotton under drought: Effects and management. *Agric. Water Manage.* **255**, 106994 (2021).

[7] Mahmood, T. et al. Insights into drought stress signaling in plants and the molecular genetic basis of cotton drought tolerance. *Cells* **9**, 105 (2019). 31906215 10.3390/cells9010105 PMC7016789

[8] Mahdy, E. E., Mohamed, H. M., Housein, M. G. & Sayed, M. A. Line× Tester Analysis for Yield and Quality Traits under Salinity Stress in Cotton (Gossypium barbadense L). *Egypt. J. Agron.* **45**, 65–80 (2023).

[9] Bhattacharya, A. Role of Plant Growth Hormones During Soil Water Deficit: A Review. In: Soil Water Deficit and Physiological Issues in Plants. Springer, Singapore. 10.1007/978-981-33-6276-5_6 (2021).

[10] Begna, T. Combining ability and heterosis in plant improvement. *Open J. Plant. Science* **6 **(1), 108–117 (2021). 10.17352/ojps.000043

[11] Mishra, S. P. et al. Breeding techniques to exploit non-additive gene action for improvement of Livestock. *Bull. Environ. Pharmacol. Life Sci.* **6**, 126–134 (2017).

[12] Bouvet, J. M., Makouanzi, G., Cros, D. & Vigneron, P. Modeling additive and non-additive effects in a hybrid population using genome-wide genotyping: prediction accuracy implications. *Heredity* **116**, 146–157 (2016). 26328760 10.1038/hdy.2015.78 PMC4806881

[13] Ghorbanzadeh, Z. et al. Integrative Genomics and Precision Breeding for Stress-Resilient Cotton: Recent Advances and Prospects. *Agronomy* **15**, 2393 (2025).

[14] Ali, K., Jilani, T. A., ul Ain, N., Ahmed, J. & Ahmed, J. Exploring Genetic Variation and Quantitative Parameters in Upland Cotton (Gossypium hirsutum L.) by Diallel Analysis. *Int. J. Agric. Innovations Cutting-Edge Res. (HEC Recognised)*. **3**, 62–74 (2025).

[15] Salama, E. A. et al. Exploring agro-morphological and fiber traits diversity in cotton (G. barbadense L). *BMC Plant Biol.* **24**, 403 (2024). 38750434 10.1186/s12870-024-04912-0 PMC11095005

[16] Sultan, M., Abdel-Moneam, M., El-Mansy, Y. & El-Morshidy, H. S. Estimating of heterosis and combining ability for some Egyptian cotton genotypes using line x tester mating design. *J. Plant. Prod.* **9**, 1121–1127 (2018).

[17] Kharche, V. P. K. & Line× Tester analysis. principles of quantitative and biometrical genetics, 101.

[18] Belay, N. Combining ability studies from line x tester mating design for grain yield and its related traits of mid-altitude maize inbred lines. *Int. J. Food Sci. Agric.* **6**, 64–75 (2022).

[19] El-Fesheikawy, A., Mahrous, H. & Baker, K. Line x Tester analysis for yield components and fiber properties in some of intra-specific cotton crosses of Gossypium barbadense L. *Res. Develop*. **32**, 923–938 (2012).

[20] Al-Hibbiny, Y. Estimation of heterosis, combining ability and gene action by using line X tester analysis in cotton (Gossypium barbadense L). *Egypt. J. Plant. Breed.* **19**, 385–405 (2015).

[21] Abdel-Aty, M. et al. Estimating the combining ability and genetic parameters for growth habit, yield, and fiber quality traits in some Egyptian cotton crosses. *BMC Plant Biol.* **23**, 121 (2023). 36859186 10.1186/s12870-023-04131-z PMC9979479

[22] Abdel-Aty, M. et al. Genetic analysis of yield traits in Egyptian cotton crosses (Gossypium barbdense L.) under normal conditions. *BMC Plant Biol.* **22**, 462 (2022). 36167520 10.1186/s12870-022-03839-8 PMC9513887

[23] Steel, R. G. D. & Torrie, J. H. Principles and procedures of statistics. (1960).

[24] Kempthorne, O. The contributions of statistics to agronomy. *Adv. Agron.* **9**, 177–204 (1957).

[25] Allard, R. The analysis of genetic-environmental interactions by means of diallel crosses. *Genetics* **41**, 305 (1956). 17247629 10.1093/genetics/41.3.305 PMC1209783

[26] Mather, K. *Biometrical genetics* (Dover publication Inc., 1949).

[27] Amer, E., Hassan, S. & Abo-Hegazy, S. Genetic analysis for yield and fiber quality traits in Egyptian cotton (Gossypium barbadense L). *Egypt. J. Plant. Breed.* **25**, 177–201 (2021).

[28] Falconer, D. S. *Introduction to quantitative genetics*Pearson Education India,. (1996).

[29] Elshazly, M., Mabrouk, A. & Soliman, A. Genetic components determination of yield and fiber quality properties in cotton (Gossypium barbadense L). *J. Plant. Prod.* **14**, 413–419 (2023).

[30] Hamed, F., Darwesh, M., Elfeki, A. E. I., Mahmoud, F. E., Abd El-Twab, R. M. & B. A. & Estimation of combining ability, heterosis, and heritability for some yield, yield components and fiber quality traits in cottons (Gossypium barbadense L). *Menoufia J. Plant. Prod.* **9**, 285–300 (2024).

[31] Al-Hibbiny, Y. & Mahmoud, B. A. Line x tester analysis for yield components and fiber properties in some cotton crosses of (Gossypium barbadense L). *Menoufia J. Plant. Prod.* **4**, 505–525 (2019).

[32] Salem, T., Rabie, H., Mowafy, S., Eissa, A. & Mansour, E. Combining ability and genetic components of Egyptian cotton for earliness, yield, and fiber quality traits. *SABRAO J. Breed. & Genetics* **52** (4), 369 (2020).

[33] Junaid, M. D. et al. Investigating the Gene Action and Combining Ability Effects on Yield Attributes in Cotton (Gossypium hirsutum L). *J. Agric. Food*. **6**, 150–164 (2025).

[34] Khan, A. et al. Coping with drought: stress and adaptive mechanisms, and management through cultural and molecular alternatives in cotton as vital constituents for plant stress resilience and fitness. *Biological research* **51**, 47 (2018). 10.1186/s40659-018-0198-z 10.1186/s40659-018-0198-z PMC6234603 30428929

[35] EL-Shazly, M., Mabrouk, A. & Soliman, A. Genetic components determination of yield and fiber quality properties in cotton (Gossypium barbadense L). *J. Plant. Prod. Mansoura Univ.* **14**, 413–419 (2023).

[36] Hussien, H. & Heterosis and combining ability for earliness yield and fiber quality characters in egyptian cotton (gossypium barbadense l.).

[37] Al-Hibbiny, Y., Ramadan, B., Max, M. S. & Heterosis and combining ability for yield and fiber quality in cotton (gossypium barbadense l.) using half diallel mating system. *Menoufia J. Plant. Production* **5**, 233–248 (2020).

[38] Darwish, S., El-Karamity, A. E., Taha, E. & Asaad, M. Stability Analyses of Early Segregating Egyptian Cotton Populations and Their Parents Across different Irrigation Intervals and Sowing Dates. *J. Plant. Prod.* **13**, 133–140 (2022).

[39] Yan, W. A systematic narration of some key concepts and procedures in plant breeding. *Front. Plant Sci.* **12**, 724517 (2021). 34603352 10.3389/fpls.2021.724517 PMC8481876

[40] El-Hashash, E. F. & El‐Absy, K. M. Smart Plant Breeding. *Custom-Designed Crop Breeding: Adv. Technol.* **1**, 359–396 (2025).

[41] Gören, H. K. & Canavar, Ö. Genetic analysis of yield and fiber quality traits in cotton (Gossypium hirsutum L.) under full and deficit irrigation conditions using full diallel method. *Euphytica* **220** (7), 115 (2024).

[42] Chattha, W. S. et al. A novel parent selection strategy for the development of drought-tolerant cotton cultivars. *Plant. Genetic Resour.* **19**, 299–307 (2021).

