# Supplementary material for: Drought stress reshapes the genetic architecture of fiber quality and developmental traits in Egyptian cotton (Gossypium barbadense L.): a line × tester analysis under contrasting irrigation regimes
Source: Sci Rep. 2026 Aug 11;16:24940. doi: 10.1038/s41598-026-63583-z (PMC13462293; doi:10.1038/s41598-026-63583-z)
Supplement: Supplementary file 1 [file 41598_2026_63583_MOESM1_ESM.docx]

## Soil and Climate Characteristics of the Experimental Site

Soil physicochemical properties were determined for the 0–30 cm and 30–60 cm depth increments of the experimental site prior to planting in both the 2024 (crossing/hybridization) and 2025 (evaluation trial) seasons (Table S1). The topsoil (0–30 cm) was clay, grading to a loam texture at 30–60 cm in both seasons. Weekly minimum, maximum, and mean air temperatures recorded throughout both growing seasons are summarized in Table S2.

**Table S1. Physicochemical properties of the experimental soil at two depths, 2024 and 2025 seasons (before planting).**

| **Property** | **2024, 0–30 cm** | **2024, 30–60 cm** | **2025, 0–30 cm** | **2025, 30–60 cm** |
| --- | --- | --- | --- | --- |
| pH (1:2.5 soil:water) | 7.90 | 7.80 | 7.11 | 7.92 |
| EC (mmhos/cm) | 0.84 | 1.34 | 0.94 | 1.29 |
| Texture class | Clay | Loam | Clay | Loam |
| Organic matter (%) | 1.30 | 1.00 | 1.41 | 1.14 |
| Total N (mg/100g) | 45.5 | 35 | 48.3 | 37 |
| CaCO₃ (%) | 6.10 | 4.80 | 5.98 | 4.32 |
| Available P (mg/100g) | 1.40 | 1.40 | 1.38 | 1.36 |
| Available K (mg/100g) | 32 | 30 | 34 | 31 |
| Available Ca (mg/100g) | 39 | 37 | 41 | 35 |
| Available Mg (mg/100g) | 11 | 10 | 10.23 | 9.98 |
| Available Na (mg/100g) | 61 | 76 | 57 | 73 |
| Available Fe (ppm) | 7.90 | 7.61 | 7.81 | 7.44 |
| Available Mn (ppm) | 4.94 | 4.63 | 4.82 | 4.51 |
| Available Zn (ppm) | 1.44 | 1.94 | 1.52 | 1.83 |
| Available Cu (ppm) | 3.05 | 3.14 | 3.12 | 3.33 |
| Available B (ppm) | 0.23 | 0.27 | 0.26 | 0.31 |

*pH: Estefan et al. (2013); EC: Estefan et al. (2013); texture: Black (1965), hydrometer method; organic matter: Black (1965), dichromate oxidation; total N: Estefan et al. (2013), semi-micro Kjeldahl; CaCO₃: Black (1965), calcimeter; available P: Olsen et al. (1954); available K: Chapman and Pratt (1978); available Fe: Lindsay and Norvell (1978); available B: Gupta (1979).*

**Table S2. Weekly minimum, maximum, and mean air temperature (°C) during the 2024 and 2025 growing seasons at the experimental site.**

| **Interval (2024)** | **Max** | **Min** | **Mean** | **Interval (2025)** | **Max** | **Min** | **Mean** |
| --- | --- | --- | --- | --- | --- | --- | --- |
| 27/4-3/5 | 30.34 | 18.66 | 24.50 | 25/4-1/5 | 30.11 | 19.03 | 24.57 |
| 4/5-10/5 | 29.15 | 21.10 | 25.13 | 2/5-8/5 | 30.07 | 19.14 | 24.61 |
| 11/5-17/5 | 30.99 | 19.15 | 25.07 | 9/5-15/5 | 31.13 | 20.42 | 25.77 |
| 18/5-24/5 | 31.94 | 18.44 | 25.19 | 16/5-22/5 | 31.97 | 19.02 | 25.50 |
| 25/5-31/5 | 28.78 | 18.68 | 23.73 | 23/5-29/5 | 34.56 | 21.81 | 28.19 |
| 1/6-7/6 | 30.03 | 21.81 | 25.92 | 30/5-5/6 | 30.29 | 20.91 | 25.60 |
| 8/6-14/6 | 29.09 | 21.19 | 25.14 | 6/6-12/6 | 33.53 | 20.96 | 27.25 |
| 15/6-21/6 | 30.77 | 21.77 | 26.27 | 13/6-19/6 | 33.50 | 22.56 | 28.03 |
| 22/6-28/6 | 31.25 | 23.86 | 27.55 | 20/6-26/6 | 33.92 | 23.11 | 28.52 |
| 29/6-5/7 | 33.55 | 24.22 | 28.88 | 27/6-3/7 | 33.37 | 22.44 | 27.91 |
| 6/7-12/7 | 35.25 | 24.28 | 29.77 | 4/7-10/7 | 34.74 | 23.26 | 29.00 |
| 13/7-19/7 | 34.40 | 24.42 | 29.41 | 11/7-17/7 | 33.77 | 23.68 | 28.73 |
| 20/7-26/7 | 33.70 | 23.74 | 28.72 | 18/7-24/7 | 34.47 | 23.86 | 29.17 |
| 27/7-2/8 | 34.47 | 23.80 | 29.13 | 25/7-31/7 | 35.04 | 24.25 | 29.65 |
| 3/8-9/8 | 34.69 | 24.57 | 29.63 | 1/8-7/8 | 34.60 | 24.84 | 29.72 |
| 10/8-16/8 | 36.33 | 24.17 | 30.52 | 8/8-14/8 | 34.46 | 24.51 | 29.49 |
| 17/8-23/8 | 33.68 | 24.06 | 28.87 | 15/8-21/8 | 33.63 | 23.81 | 28.72 |
| 24/8-30/8 | 34.82 | 24.56 | 29.69 | 22/8-28/8 | 33.90 | 24.14 | 29.02 |
| 31/8-6/9 | 29.39 | 20.90 | 25.15 | 29/8-4/9 | 29.29 | 21.49 | 25.39 |
| 7/9-13/9 | 31.95 | 23.41 | 27.68 | 5/9-11/9 | 34.07 | 24.24 | 29.16 |
| 14/9-27/9 | 31.87 | 22.77 | 27.32 | 12/9-18/9 | 32.20 | 23.39 | 27.80 |
| 21/9-27/9 | 31.52 | 21.93 | 26.72 | 19/9-25/9 | 32.19 | 22.66 | 27.43 |
| 28/9-4/10 | 30.58 | 20.52 | 25.55 | 26/9-2/10 | 33.27 | 22.61 | 27.94 |
| 5/10-11/10 | 29.71 | 20.29 | 25.00 | 3/10-9/10 | 30.30 | 22.69 | 26.50 |
| 12/10-18/10 | 29.14 | 21.43 | 25.29 | 10/10-16/10 | 28.80 | 20.96 | 24.88 |
| 19/10-25/10 | 27.21 | 21.29 | 24.25 | 17/10-23/10 | 28.73 | 20.44 | 24.59 |
| 26/10-1/11 | 30.14 | 18.14 | 24.14 | 24/10-30/10 | 28.94 | 19.88 | 24.41 |
| 2/11-8/11 | 28.15 | 18.07 | 23.11 | 31/10-6/11 | 27.89 | 19.64 | 23.77 |

*Temperatures are means of daily maxima, minima, and means over each seven-day interval, recorded at the nearest meteorological station to the experimental site.*
